# Linking innovation with hospital performance: the mediating role of experiential healthcare marketing

**DOI:** 10.3389/frhs.2026.1875713

**Published:** 2026-07-21

**Authors:** Abdul Rauf, Lilian Anthonysamy, Hamid Mahmood, Norhilmi Muhammad, Muhammad Hammad, Ihtisham Ullah

**Affiliations:** 1 Centre for Management and Marketing Innovation, COE of Business Innovation and Communication, Multimedia University, Cyberjaya, Malaysia; 2 Centre for Management and Marketing Innovation, COE of Business Innovation and Communication, Faculty of Management, Multimedia University, Cyberjaya, Malaysia; 3 Department of Management Sciences, TIMES University, Multan, Pakistan; 4 Department of Nationality and Entrepreneurship, Faculty of General Studies and Advanced Education, Universiti Sultan Zainal Abidin, Kuala Nerus, Malaysia; 5 Faculty of Business Economics and Social Development, Universiti Malaysia Terengganu, Kuala Nerus, Malaysia; 6 Strategic Research Institute, Asia Pacific University of Technology and Innovation, Kuala Lumpur, Malaysia

**Keywords:** entrepreneurial innovation, healthcare, hospital performance, service innovation, technological innovation

## Abstract

**Background:**

In the rapid evolution of the healthcare sector, innovation is recognized as an important factor in hospital performance; however, the mechanisms by which types of innovation translate into performance remain unexplored. Therefore, current study examines the effects of entrepreneurial, technological, and service innovation on hospital performance, with experiential healthcare marketing as a mediator and patient trust as a moderator.

**Methods:**

The data were collected from 340 patients at private hospitals using a self-administered questionnaire. Structural equation modeling was performed for path analysis using SmartPLS, and statistical measures were analyzed using SPSS (25.0).

**Results:**

The present study results revealed that technological innovation and entrepreneurial innovation increase hospital performance directly and through experiential healthcare marketing. Surprisingly, relationship between service innovation and experiential healthcare marketing is not significant, as service innovation is often process-oriented and focuses on the delivery system, whereas experiential marketing is intrinsically emotional and perception-based; therefore, it may fail to deliver meaningful experiential outcomes. Additionally, moderating effect of patient trust is also not significant, indicating that experiential marketing operates independently in current context.

**Discussion:**

This study underscores a critical gap in digital transformation by proposing an integrative model that explores how innovative drivers affect hospital performance through patient-centered mechanism. This study also examines mediating role of experiential marketing a under research variable in healthcare context.

## 1 Introduction

In this digital transformation era, healthcare organizations face immense pressure to provide patient-centered care, digitalize operations, and maintain competitive environment. Therefore, digital transformation becomes a necessity in healthcare, driven by systematic inefficiencies, increasing patient expectations, and technological disputes (1). However, numerous hospitals struggle to transform innovation into performance improvements. Hence, the current study examines three crucial innovative drivers: service innovation, which reconceptualizes experience with personalization, value co-creation, and digital empowerment (2); entrepreneurial innovation that enabled hospitals to adopt opportunity driven change and disruptive health solutions (3); and technological innovation that consist on AI adoption in diagnostics, telemedicine's platforms, digital tools, and e-health records (4).

Healthcare innovation is often overlooked by patients and requires engagement to be acknowledged (5). This disconnect may create persistent problem, result in patient dissatisfaction, mistrust, and low engagement despite technological improvements (6). These issues also threaten the long-term growth of digital transformation and feature strategic failures in patient experience (7). In this regard, experiential marketing can play an important mediating role in promoting digital transformation among patients by building trust and designing interactive, emotionally profound experiences.

Further, the effectiveness of experiential marketing heavily depends on psychological factors in determining whether digital innovation is resisted or embraced (8). Additionally, patient trust as an important psychological factor can moderate the relationship among experiential marketing and hospital performance. Patient trust also plays a fundamental role in the digital health environment, where patients have concerns about data privacy and impersonal care (9). The extent studies isolate the digital transformation in strategic marketing (10, 11) and miss the complex interplay among technological changes and patient experience. This study explores experiential marketing, an underutilized strategy in the healthcare sector that may enhance the patient experience.

This study provides several theoretical contributions to advance healthcare management literature. First, it proposes an integrative model that explores how innovative drivers affect hospital performance through patient-centered mechanism. Second, this study extends exiting literature on innovation by integrating entrepreneurial innovation, service innovation and technological innovation in a single framework as past studies examines these dimensions in isolation. Third, current study proposed mediated-moderated framework to link digital transformation (entrepreneurial innovation, technological innovation, service innovation), experiential healthcare marketing, hospital performance and patient trust to understand digital transformation outcomes in healthcare. These contributions mutually advance theoretical understanding on how innovation interact with patient experience and influence hospital performance and provide deep understanding of value creation in healthcare organization. Without understanding this mechanism, healthcare service providers struggle to create a sustainable, effective, and ethical marketing strategy that contributes to hospital performance.

## 2 Literature review

### 2.1 Entrepreneurial innovation and experiential healthcare marketing

Entrepreneurial innovation in healthcare sector emerged as an important factor in delivering services through new technological solutions and encountered challenges in service delivery (12). It also provides opportunities for entrepreneurial innovation to bridge gap with traditional healthcare systems while forming sustainable businesses (13). This transformation refers to the essential shift in healthcare organization approaches regarding operational efficiency and patient care. Further, the adoption of entrepreneurial innovation may improve patient satisfaction, reduce errors, enhance team coordination, and improve overall patient experience (14). Entrepreneurial innovation can also play an important role in patient-centered experience beyond conventional promotional tactics (15), through experiential marketing. Entrepreneurial health service providers can also enhance the patient experience through experiential marketing tactics by providing hands-on interaction that creates an emotional connection and builds trust. Therefore, we hypothesized that,.

**H1:** Entrepreneurial Innovation has positive impact on experiential marketing.

### 2.2 Technological innovation and experiential healthcare marketing

Technological innovations refer to the provision of new services/products and to the improvement of existing services/products in healthcare through new technologies (16). It also predicts that technological innovation could improve healthcare outcomes, enhance healthcare quality, and improve efficiency, which would be favorable for healthcare organizations and patients (17). Technological innovation redefined efficiency, quality of healthcare service delivery, and patient experience. Technological innovation in service-linked tools like telemedicine to reduces patient waiting times, expands platform access (18), maintain electronic health records and improve coordination among departments (19), and mobile applications increase patient engagement and provide self-service management (20). These technological advancements create memorable, engaging and emotionally resonant experiences with patients. Therefore, the current study hypothesizes that,

**H2:** Technological innovation positively impact experiential marketing.

### 2.3 Service innovation and experiential healthcare marketing

Service innovation in the healthcare sector is illustrated by the adoption of unique service processes, patient-centered solutions, and service delivery models that outstrip traditional care methods (21). It also used as a strategic approach to increase customer experience and hospital performance. The studies found that service innovation focused on personalization and improved quality is critical for health services (22, 23). Innovation such as mobile health apps (24), remote monitoring, and home-based services (25), improve price transparency, overall service efficiency (26), and decentralized healthcare services moving from hospital-centric to patient-inclusive. Furthermore, service innovation in healthcare sector foster value co-creation by involving patients' decisions in treatment process (27). It also plays an important role in determining experiential marketing within healthcare sector. Service innovation can transform existing services into a personalized, interactive and engaging experience (28). Patients memorable experience can further strong emotional connection and strengthen trust in service delivery process. Therefore, we argue that service innovation has a strong foundation in experiential marketing practices.

**H3:** Service innovation positively impact experiential healthcare marketing.

### 2.4 Experiential healthcare marketing and hospital performance

Experiential marketing highlights the meaningful, emotional and memorable customer experience during their interaction (29). It differs from traditional marketing and focuses on emotions, cognitions, senses, and behaviors that influence customer perception (30). Experiential marketing in a healthcare setting manifests as personalized care, empathetic communication, the incorporation of new technologies, and a comfortable environment that increases patient engagement (31). This experience plays important role in patient trust, loyalty and hospital performance. Experiential marketing strengthens the relationship between the service provider and the customer (32), which ultimately influences hospital performance. The positive, emotionally fulfilling, a personalized experience enhance patient satisfaction, increase competitiveness and reputation (33). Therefore, we argue that experiential marketing in the healthcare sector could be a crucial factor in hospital performance, as it increases hospital capabilities and fulfills patient experiential expectations.

**H4:** Experiential healthcare marketing positively impact hospital performance.

### 2.5 Experiential healthcare marketing as mediator

Experiential healthcare marketing crafts an emotional, meaningful, and rational experience for patients during their healthcare journey. It also recognizes that patients evaluate hospitals not only on clinical outcomes but also on experience and care delivery (34). The studies reveal that positive experiences increase trust and loyalty, which enhance hospital performance (35, 36). In parallel, digital transformation (service, technological, and entrepreneurial innovation) has become an important factor in healthcare, enabling efficient processes and personalized services. However, the benefits of digital transformation are not always realized, and patients do not value or fully perceive service and technology innovations unless they are embedded in their healthcare experience (37). Therefore, current study uses experiential marketing as mediator in between entrepreneurial innovation, service innovation, technological innovation and hospital performance to translate digital transformation into hospital performance. The digital transformation increases hospital outcomes (38), and experiential values influence performance outcomes (39). It also shapes patients' cognitive, emotional, and rational responses to innovative healthcare. Further, in the absence of experiential engagement digital transformation remain operational but limit performance outcomes. Thus, experiential healthcare marketing act as a bridge between digital transformation and hospital performance by ensuring innovative outcomes are positively interpreted and experienced by patients.

**H5a:** Experiential healthcare marketing mediates the relationship between **e**ntrepreneurial innovation and hospital performance.

**H5b:** Experiential healthcare marketing mediates the relationship between technological innovation and hospital performance.

**H5c:** Experiential healthcare marketing mediates the relationship between service innovation and hospital performance.

### 2.6 Patient trust as moderator

Patient trust is a fundamental factor in healthcare sector and plays a crucial role in organizational performance and patient service encounters (40). It refers to the patient's belief regarding hospital competencies, transparency, and reliability, particularly in uneven and unequal healthcare context (41). The studies highlighted that trust impacts patients' willingness to engage with service providers, follow service providers' recommendations, and maintain long-term relationships (42, 43). Patient trust is a crucial factor in healthcare experiential marketing and determines how experiential stimuli are internalized and interpreted. The high level of trust among patients increases service interaction, makes patients more responsive to experiential cues, and increases favorable intention towards healthcare service providers (43). This increase in experiential marketing efficiency enhances emotional attachment and boosts behavioral outcomes that contribute to hospital performance. In contrast, a low level of trust weakens experiential initiatives' impact, resists engagement, and leads to cautious interpretation of experience (44). Additionally, trust decreases uncertainty and perceived risk (45), allowing experiential strategy to influence hospital performance (46). Experiential marketing also impacts behavioral responses and emphasizes experience-driven healthcare management. Therefore, we assume that patient trust moderates the relationship between experiential marketing and hospital performance.

**H6:** Patient trust moderates the relationship between experiential marketing and hospital performance.

**Figure 1:**
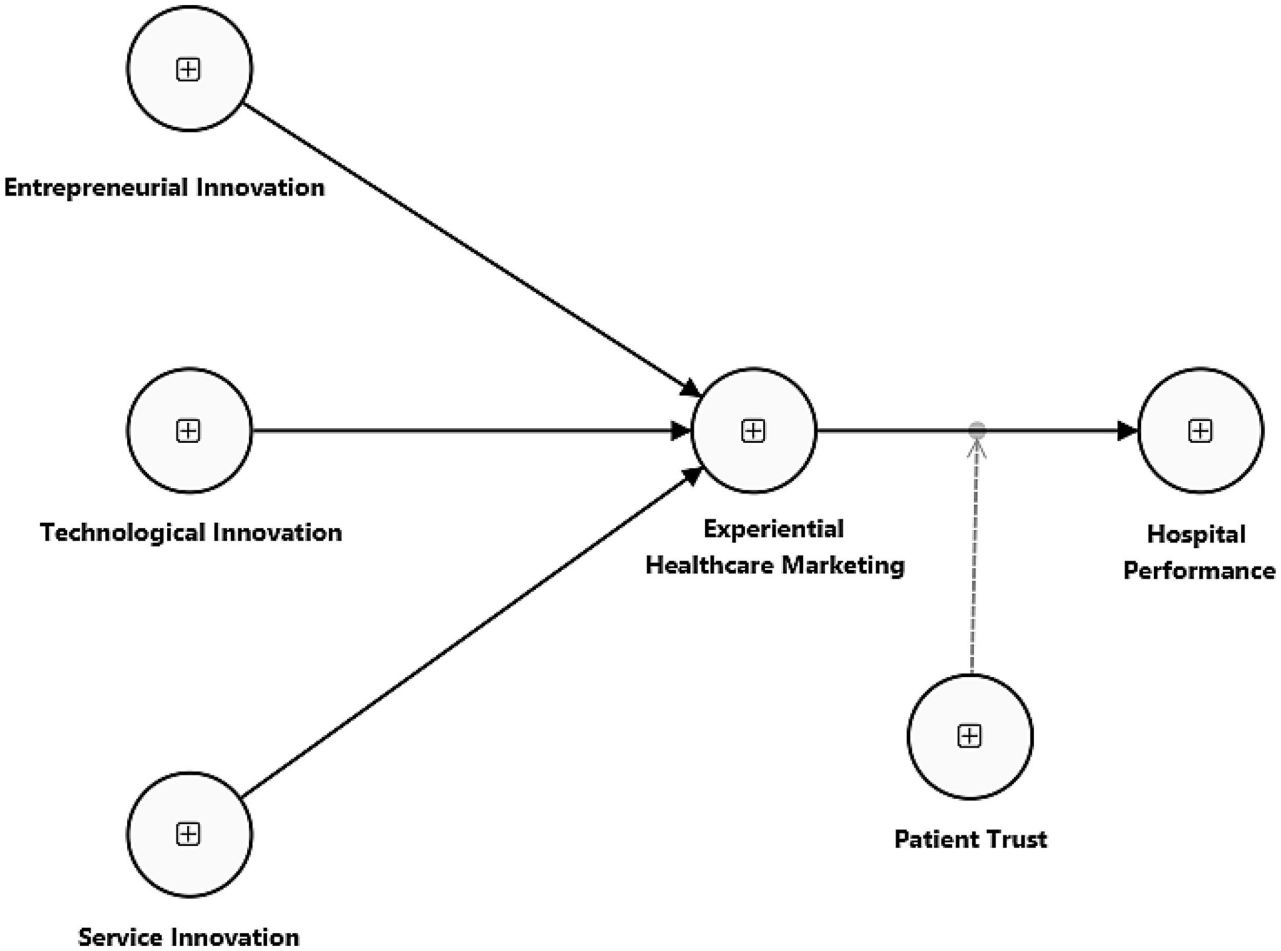
Conceptual framework. Source: Authors' own work (SmartPLS).

Combining all hypotheses, the proposed research framework is presented in Figure 1.

## 3 Methodology

This study used a quantitative, cross-sectional research design, with a structured, self-administered questionnaire for data collection and hypothesis testing. The research sample included patients (users of healthcare services) with recent hospital service experience, and the unit of analysis was the individual respondent who rated the innovation-based practices, the experience of exposure to experiential healthcare marketing, trust, and perceived hospital performance based on their experience in healthcare.

### 3.1 Data collection

Non-probability convenience sampling was used to collect data due to the difficulty for researchers to collect data from entire population (47). The data were collected from 5 major private hospital in Lahore, Punjab. The province Punjab were selected because it is the biggest province in Pakistan by population and Lahore is key center of healthcare service in the region. Data collection was conducted between February 20, 2026, and March 5, 2026, using a self-administered questionnaire distributed both in person and via an online form. The participants were patients and aged 20 years or above who recently experienced services were approached. To enhance response quality, the questionnaire included a brief screening question to ensure respondents had received services at the hospitals. All the participants were informed prior data collection about study purpose, right to withdraw and voluntary participation. The written informed consent was obtained from the participants while data collected in-person and consent statement were presented in the beginning of the questionnaire for online participants. All the responses were confidential and used for academic research purpose only. Three hundred and forty responses remained to be analyzed.

### 3.2 Procedural CMV control

Common method variance is a possible issue that could arise when the questionnaire data is collected from one individual. To minimize the possibility of CMV, we used several procedural remedies during data collection process. Every respondent was assured about their voluntary participation and that their responses would remain confidential and anonymous. This remedy was intended to collect and encourage honest responses. Secondly, the questionnaire was also designed using clear and simple wording for better understanding. Lastly, the items measuring different constructs were presented in separate sections in the questionnaire so that the respondents could clearly distinguish between variables. These steps helped reduce the likelihood that the observed variables were caused by the measurement method rather than by the theoretical relationships.

### 3.3 Instrument

All constructs were measured using multi-item scales adapted from established literature and contextualized to the healthcare setting. The measurement instrument was developed by adapting previously validated scales. Entrepreneur Innovation (EI) was measured using 9 items adapted (48), while 5-items Patient Trust (PT) scale were adapted (49). The five items for Technological Innovation (TI) and 6 items for Service Innovation (SI) were adapted from validated studies (50). 10 items used to measure Experiential Healthcare Marketing (EHM) were adapted (51). Hospital Performance (HP) was measured using 9 items adapted (52). All items were measured on a five-point Likert scale where 1 represents strongly disagree and 5 represents strongly agree and refined for clarity and relevance to hospital services. The total number of questionnaire items were 44 items. The questionnaire comprised two sections: (i) demographic/profile information, and (ii) measurement items for latent variables.

### 3.4 Data analysis method

The hypothesized framework was tested using Partial Least Squares Structural Equation Modelling (PLS-SEM) in SmartPLS 4. Bootstrapping with 5,000 resamples was applied to evaluate the significance of path coefficients, indirect effects, and moderation effects. The measurement model was evaluated to achieve reliability and validity. The reliability of the indicators was assessed by standardized outer loadings; internal consistency reliability was assessed with Cronbach's alpha and Composite Reliability; convergent validity was assessed with AVE; and discriminant validity was assessed with the HTMT ratio. Since data were collected using a single questionnaire, common method bias was assessed using a collinearity-based approach by examining the inner VIF values. Once the measurement model's sufficiency was established, the structural model was tested using bootstrapping, the coefficient of determination (R^2^), and tests of mediation and moderation. To evaluate mediation, the impact of EI, TI, and SI on Hospital Performance was estimated by analyzing the indirect effects of EHM, and moderation was estimated using an interaction term (PT × EHM) predicting Hospital Performance. The ethical approval was obtained from the Research Ethics Committee of Multimedia University, Malaysia, with reference no. RMC/REC/EA/005/2026, dated 03/02/2026.

## 4 Data analysis and results

The data were analyzed using SmartPLS and a two-step procedure, that is, the measurement model was evaluated followed by the structural model. The collected data go through a thorough screening process, and 340 usable responses comprised the final sample for the study.

### 4.1 Demographic analysis

The respondent profile shows that 62% of participants were male and 38% were female. Most respondents belonged to the age group of 27–34 years (32%), followed by 41 years and above which were (27%), 34–41 (22%), and 20–27 (19%). Regarding income, the majority of the respondents stated the earnings of RS. 80,000–Rs. 100,000 PKR (43%), followed by Rs. 60,000–Rs. 80,000 PKR (27%), 17% were those who were getting less than Rs. 60,000/-, and people who were getting more than or equal to Rs. 100,000 PKR were 13%. In terms of marital status, 45% were married, 21% were single, 18% were divorced, and 16% were widowed. Most respondents reported using government hospitals (61%), while teaching hospitals (12%) and other hospitals (27%) were also represented. Finally, hospital visits were distributed as once (36%), twice (29%), thrice (19%), and thrice or more (16%), indicating varying levels of exposure to hospital services (see Table 1).

**Table 1:** Demographic profile (340).

Demographic Variable	Category	Frequency (*n*)	Percentage (%)
Gender	Male	211	62
	Female	129	38
Age Group	20–27	65	19
	27–34	109	32
	34–41	75	22
	41 or above	91	27
Income	Less than 60,000 PKR	58	17
	60,000–80,000 PKR	92	27
	80,000–100,000 PKR	146	43
	100,000 PKR or above	44	13
Marital Status	Single	71	21
	Married	153	45
	Divorced	61	18
	Widow	55	16
Nature of Hospital	Government	207	61
	Teaching	41	12
	Others	92	27
Hospital Visits	Once	122	36
	Twice	99	29
	Thrice	65	19
	Thrice or more	54	16

Source: Authors own work.

### 4.2 Measurement model

The outer loadings were used to assess the reliability indicators. In general, the retained indicators contributed moderately to the constructs, indicating the validity of the measurement model. The Cronbach alpha and composite reliability were used to establish internal consistency and reliability. The findings showed a high level of reliability for most constructs, with Cronbach's alpha values of 0.900 (EHM), 0.886 (EI), 0.880 (Hospital Performance), 0.778 (SI), and 0.710 (TI). Internal consistency was also supported using composite reliability (rho c), with values of 0.917, 0.908, 0.903, 0.792, 0.884, and 0.849. Average Variance Extracted (AVE) was used to assess convergent validity. EHM (0.527), EI (0.523), Hospital Performance (0.511), PT (0.506), SI (0.559), and TI (0.529) exceeded the recommended minimum threshold of 0.50. Table 2 presents the reliability and validity values.

**Table 2:** Reliability and validity.

Construct	Items	Loadings	Alpha	CR	AVE
Experiential Health Marketing	EHM1	0.752	0.900	0.917	0.527
	EHM2	0.715			
	EHM3	0.783			
	EHM4	0.753			
	EHM5	0.654			
	EHM6	0.765			
	EHM7	0.718			
	EHM8	0.630			
	EHM9	0.716			
	EHM10	0.758			
Entrepreneurial Innovation	EI2	0.707	0.886	0.908	0.523
	EI3	0.666			
	EI4	0.733			
	EI5	0.740			
	EI6	0.701			
	EI7	0.732			
	EI8	0.746			
	EI9	0.753			
	EI1	0.724			
Hospital Performance	HP1	0.713	0.880	0.903	0.511
	HP2	0.716			
	HP3	0.739			
	HP4	0.701			
	HP5	0.776			
	HP6	0.757			
	HP7	0.723			
	HP8	0.600			
	HP9	0.693			
Patient Trust	PT1	0.620	0.710	0.792	0.506
	PT2	0.740			
	PT3	0.664			
	PT4	0.534			
	PT5	0.723			
Service Innovation	SI1	0.762	0.843	0.884	0.559
	SI2	0.746			
	SI3	0.744			
	SI4	0.722			
	SI5	0.713			
	SI6	0.797			
Technological Innovation	TI1	0.701	0.778	0.849	0.529
	TI2	0.687			
	TI3	0.773			
	TI4	0.741			
	TI5	0.732			

Source: Authors own work.

The HTMT criterion was applied to assess discriminant validity. It shows that all items measure their own latent construct and they discriminate from other latent variables. Overall, the HTMT values for all variables fell within acceptable ranges, showing acceptable discriminant validity as shown in Table 3.

**Table 3:** HTMT.

	EHM	EI	Hospital performance	PT	SI	TI	PT × EHM
EHM							
EI	0.587						
Hospital Performance	0.481	0.522					
PT	0.569	0.681	0.646				
SI	0.479	0.627	0.473	0.850			
TI	0.721	0.698	0.734	0.805	0.750		
PT x EHM	0.072	0.144	0.057	0.238	0.158	0.240	

EHM, experiential health marketing; EI, entrepreneurial innovation; PT, patient trust; SI, service innovation; TI, technological innovation.

Source: Authors own work.

### 4.3 Collinearity assessment

Collinearity was examined using inner-model VIF values to ensure that multicollinearity was not inflating standard errors. The VIF results were low and well within acceptable limits as shown in Table 4.

**Table 4:** Collinearity.

Paths	VIF
EHM → Hospital _Performance	1.277
EI → EHM	1.661
PT → Hospital _Performance	1.330
SI → EHM	1.737
TI → EHM	1.830
PT × EHM → Hospital _Performance	1.047

Source: Authors own work.

The inner-model VIF values ranged from 1.047 to 1.830, indicating that multicollinearity was not a concern in the structural model. Since all variables were measured using a single self-reported questionnaire, additional steps were taken to assess the possibility of common method variance. Procedurally, anonymity, confidentiality, voluntary participation, and clear item wording were used to reduce response bias. The single factor test and the full collinearity (VIF) were analyzed statistically by Harman's single factor test. The first factor accounted for less than the recommended threshold of 50% of the total variance (38.729%) and VIF values of the full collinearity were less than the recommended limit of 3.3 (range 1.718 to 2.633). These results indicated that common method variance had little or no impact on the results.

### 4.4 Structural model assessment and hypothesis testing

**Figure 2:**
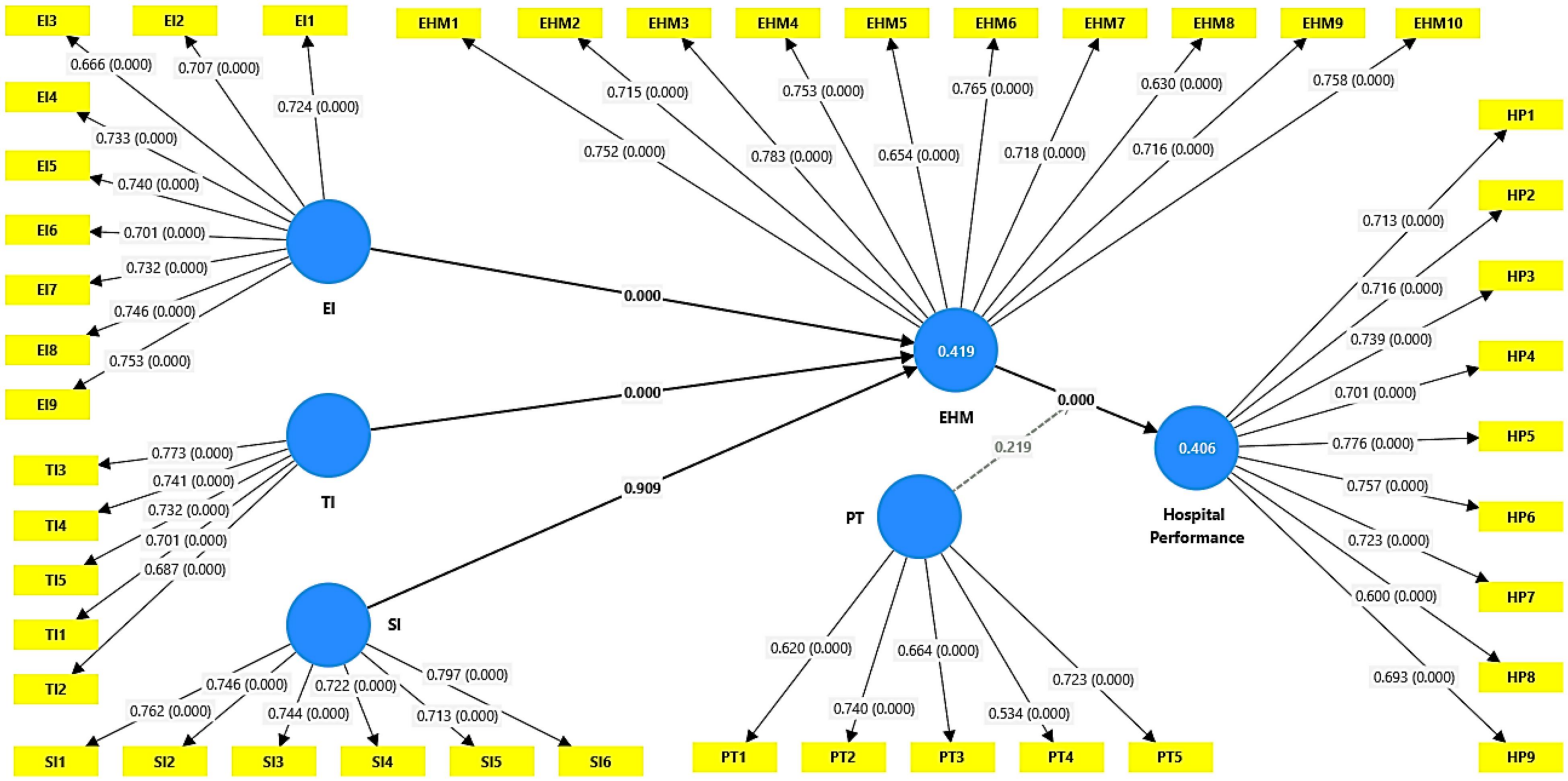
Structural model. Source: Authors own work (SmartPLS).

The structural model was assessed using bootstrapping of 5,000 resamples based on 340 valid responses and the hypotheses were evaluated using standardized coefficients (*β*), *t*-statistics, and *p*-values. The model demonstrates acceptable explanatory power, accounting for 41.9% of the variance in EHM (R^2^ = 0.419) and 40.6% of the variance in Hospital Performance (R^2^ = 0.406), indicating moderate predictive capability for the proposed framework in Figure 2. The results show that EHM has a is positively and significantly associated with on Hospital Performance (*β* = 0.196, *t* = 3.542, *p* = 0.000), indicating that stronger experiential healthcare marketing is associated with higher perceived hospital performance. Regarding the antecedents of experiential healthcare marketing, Entrepreneur Innovation shows a positive and significant association with EHM (*β* = 0.263, *t* = 4.477, *p* = 0.000), whereas TI also shows a stronger positive association with EHM (*β* = 0.454, *t* = 7.723, *p* = 0.000). Conversely, EHM is not significantly predicted by SI (*β* = 0.006, *t* = 0.114, *p* = 0.909), indicating that service innovation, at least as reflected in this set of data, is not significantly related to stronger experiential healthcare marketing. The model also shows that performance shows significant direct associations of constructs relating to innovation. There is a positive and significant association between EI and Hospital Performance (*β* = 0.052, *t* = 2.706, *p* = 0.007) and TI is also positively and significantly associated with Hospital Performance (*β* = 0.089, *t* = 2.886, *p* = 0.004). On the other hand, SI is not significantly associated with Hospital Performance (*β* = 0.001, *t* = 0.109, *p* = 0.913). It is worth noting that PT is strongly and significantly associated with Hospital Performance (*β* = 0.532, *t* = 8.939, *p* = 0.000), indicating that patient trust is one of the determinants of hospital performance perception in the proposed framework (see Figure 2).

### 4.5 Moderation test

The moderating role of PT on the relationship between EHM and Hospital Performance was tested via the interaction term PT × EHM → Hospital Performance. The interaction effect was positive but not statistically significant (*β* = 0.077, *t* = 1.228, *p* = 0.219). This implies that the impact of experiential healthcare marketing on hospital performance does not significantly differ across levels of patient trust in this sample. In other words, while both EHM and PT independently contribute to Hospital Performance, the strengthening (or weakening) effect of PT on the EHM–performance relationship is not supported (see Table 5).

**Table 5:** Structural model results.

Hypotheses	*β*	T statistics	*P* values	Decision
EI → EHM	0.263	4.477	0.000	Supported
TI → EHM	0.454	7.723	0.000	Supported
SI → EHM	0.006	0.114	0.909	Not Supported
EHM → Hospital Performance	0.196	3.542	0.000	Supported
PT × EHM → Hospital Performance	0.077	1.228	0.219	Not Supported
EI → EHM → Hospital _Performance	0.052	2.706	0.007	Supported
SI → EHM → Hospital _Performance	0.001	0.109	0.913	Not Supported
TI → EHM → Hospital _Performance	0.089	2.886	0.004	Supported

Source: Authors own work.

### 4.6 Mediation test

The mediating role of EHM was examined through bootstrapped specific indirect effects. The indirect effect of Entrepreneurial Innovation on Hospital Performance via EHM is positive and significant (EI → EHM → Hospital Performance: *β* = 0.052, *t* = 2.706, *p* = 0.007), indicating that experiential healthcare marketing explains the association between entrepreneurial innovation on hospital performance. Similarly, the indirect effect of Technological Innovation on Hospital Performance through EHM is positive and significant (TI → EHM → Hospital Performance: *β* = 0.089, *t* = 2.886, *p* = 0.004). However, the indirect pathway for Service Innovation is not significant (SI → EHM → Hospital Performance: *β* = 0.001, t = 0.109, *p* = 0.913), which is consistent with the non-significant effect of service innovation on EHM. Overall, the results support the central mechanism in which entrepreneurial and technological innovation are associated with higher perceived hospital performance partly through strengthening experiential healthcare marketing, whereas service innovation does not exhibit a meaningful direct or indirect effect in the tested model.

## 5 Discussion on findings

The results of this study show that entrepreneurial innovation is positively and significantly associated with experiential healthcare. This suggests that hospitals with stronger entrepreneurial approaches, such as opportunity-driven initiatives and flexible operational strategies to create meaningful patient experiences. The findings of the current study are consistent with prior studies, which indicate that entrepreneurial innovation motivates healthcare organizations to move away from standard service delivery and toward patient-centered value creation (53). It also aligns with previous literature, which emphasizes the role of entrepreneurial thinking in fostering creativity, adaptability, and responsiveness within healthcare systems (54). Consequently, hospitals that adopt entrepreneurial practices may be linked with stronger experiential marketing by offering more interactive and personalized healthcare services.

The study further shows that technological innovation is positively associated with experiential healthcare marketing. The current study result aligns with existing research on technologies such as telemedicine electronic records and digital healthcare platforms that are more efficient (55). These technological tools may support better communication, shorten wait times, and offer patients more convenient access to healthcare services. The results reinforce other extant studies that propose technological advancements are linked with improved patient interaction and service quality (56, 57). By leveraging digital technologies in healthcare operations, hospitals may be better positioned to create memorable, interactive experiences for their patients. Contrary to expectations, the results show that service innovation does not have a significant impact on the experiential healthcare marketing and hospital performance.

The findings suggest that introducing service innovation alone in the healthcare sector is not sufficient to shape patients' experiential perceptions. Although, service innovation recognized as an important factor in organizational performance, but its impact in healthcare sector often indirect and preliminary depends on actual experience and service process. In current context, service innovation initiatives in studied hospitals may still in early stage or preliminary process oriented instead of visible innovation. Moreover, structural or operational constrains may limit the association between service innovation and performance outcomes. Additionally, service innovation is often process-oriented and focuses on the delivery system, whereas experiential marketing is intrinsically emotional and perception-based; therefore, it may fail to deliver meaningful experiential outcomes. Patients do not fully perceive the innovation's impact unless it is effectively communicated (58).

The results also support a positive association between experiential healthcare marketing and hospital performance. Hospitals that focus on developing meaningful, emotionally pleasing patient experiences are more likely to be associated with higher levels of patient satisfaction (59), loyalty, and a positive reputation. Experiential marketing focuses on care tailored to the individual, empathetic communication, and patient participation in the service process. The findings of the present study are consistent (60), who stated that when patients feel valued and engaged during their healthcare journey, they are more likely to trust the healthcare institution and recommend its services to others.

The moderating role of patient trust in the relationship between experiential healthcare marketing and hospital performance was also not supported. This finding shows that the effectiveness of experiential marketing does not depend on the level of patient trust in the healthcare context. In current context, trust act as baseline expectation rather than differentiating factor. Patients characteristically assume a fundamental level of trust in healthcare service providers due to the high-stakes nature. Additionally, non-significant moderating effect may attribute to restively stable as there are very less variation of respondents in healthcare context. In private hospitals, trust often pre-established and limit variability among respondents resulting constrain moderating potential. Consequently, we also argue that trust may strongly function as direct predictor of patient satisfaction and behavior rather than boundary condition. This leads to the conclusion that, trust is an independent factor that is associated with hospital performance, does not necessarily strengthen the relationship between experiential marketing and performance in the studied context.

### 5.1 Theoretical contributions

This study contributes to the literature on experiential healthcare marketing in an important and timely way by building upon the literature on entrepreneurial innovation and technological innovation in the healthcare sector. Previous studies have mainly investigated experiential marketing in the retail and service industries; however, few have examined its use in the healthcare sector (8). The research explores the development of a conceptual framework for entrepreneurial, service, and technological innovation in relation to experiential healthcare marketing and hospital performance. This integrated model is useful for understanding the contributions of various forms of innovation to value creation in healthcare organizations. By bringing together knowledge from innovation management and marketing theory, the research offers a more holistic approach to how hospitals can improve organizational outcomes with an innovative marketing program.

This study also contributes by highlighting the mediating role of experiential healthcare marketing in the relationship between innovation and hospital performance. Prior research has focused on direct link between innovation and organizational performance; however, understanding the mechanisms through which innovation is linked with performance in healthcare settings is poorly developed. The results revealed that experiential marketing serves as an important factor in innovative practices which associated with stronger patient engagement and perceived hospital performance. This insight adds value to the theoretical discussion by highlighting experiential marketing as a key connection between innovation capabilities and performance results. Furthermore, this research advances theoretical understanding and examine the role of patients' trust in healthcare performance. Trust has been extensively studied in healthcare literature but scholarly work on how trust interacts with experiential marketing strategies is scarce.

### 5.2 Practical implications

This study has several significant implications for the practice of healthcare managers and hospital administrators. First, this paper highlights the importance of entrepreneurial innovation in enhancing the experiential marketing in healthcare. The hospital leaders should encourage an entrepreneurial culture, which promotes creativity, perception of opportunities and innovative solutions. The hospitals can also create innovative strategies for engaging with patients and delivering services by promoting healthcare professionals. This can help healthcare organizations to create more interactive and personalized patient experiences. Further, this study contributes in technological innovation with experiential healthcare marketing and suggest that institutions should invest in existing digital technologies (61). The healthcare technology can enhance the efficiency of services and patient interactions, including telemedicine platforms, electronic health records, mobile health applications, whereas AI-driven diagnostic systems increase clinical decision making and improve transparency. As a result, these technologies provide opportunity to enhance patient convenience, shorten wait times, and extend healthcare access. Thus, it is important for healthcare managers to prioritize their digital transformation strategies to improve patient experience and outcomes. Another significant implication is the contribution of experiential healthcare marketing to enhance hospital performance. It is not enough for healthcare organizations to execute traditional healthcare promotional tactics and they should provide patient-centric experiences on the healthcare journey. The hospitals can enhance their patients' satisfaction and loyalty by prioritizing the experiential and emotional elements (62). Last but not least, the findings related to the non-significant impact of service innovation give valuable insights to health care managers. Hospitals should aware that new service processes don't always lead to better patient experience or better hospital performance. Rather, service innovations need to be well communicated and inbuilt in the patient experience so that patients can see the value.

## 6 Conclusion

The current study conclude that innovation plays a vital role in hospital performance and enhance overall patient experience. This study also demonstrate that entrepreneurial and technological innovation plays significant role in experiential healthcare marketing and enhance hospital performance. Experiential healthcare marketing plays important role in turning innovation into patient engagement and satisfaction. The study also validates the critical and direct relationship between patient trust and hospital performance, highlighting the importance of trust, reliability, transparency and confidence in healthcare relationships. Additionally, service innovation showed insignificant effect and indicating that service innovations do not have influential effect if patients are not evident and experiential. In sum, this research suggests that hospitals can have better performance when they integrate innovation with patient-centred and patient emotionally engaging healthcare experiences. The study offers a holistic view of the relationship of innovation, trust and experiential marketing in healthcare. It also represents a theoretical contribution, because it expands the literature of experiential marketing in the health care sector and a practical contribution, through suggestions to managers on innovative approaches which are both operationally efficient and patient oriented.

## 7 Limitations and future research

The current study is cross-sectional which limits the ability to establish causal relationships among entrepreneurial innovation, technological innovation, service innovation, experiential healthcare marketing, patient trust, and hospital performance. Data were collected from five private hospital in Lahore using convenience sampling technique which restrict generalizability of the findings. Future studies should employ probability sampling and collect data from a number of public, private, and teaching hospitals across various regions to enhance generalizability. Moreover, the research was largely based on a single questionnaire-based instrument which would not necessarily provide a complete picture of the complexity of hospital performance and innovation practices. These issues need to be taken into consideration when interpreting the results and applying them to other healthcare settings.

Further, this research focused on selected dimensions of innovation, entrepreneurial innovation, technological innovation, and service innovation; which other inductors relevant to innovation like leadership support, organizational culture, staff competence, regulatory pressure as well as digital readiness were not incorporated into the model. Similarly, hospital performance was measured from the patient's perspective rather than institutional objective measures. This may not entirely capture other dimensions such as operational efficiency, financial outcomes, service quality, or clinical performance. The future studies can adopt multisource approach by incorporating objective and subjective performance measures to provide more inclusive evaluation of hospital performance. The findings also revealed that service innovation did not have a significant impact on experiential healthcare marketing or hospital performance, which could reflect a measurement limitation or the idea that patients do not easily recognize internal service improvements. In the same way, the mediating effect of patient trust was not supported, suggesting that other boundary conditions better explain when experiential healthcare marketing leads to higher performance. Future studies should address these limitations by adopting longitudinal or mixed-methods designs to more adequately examine causal effects and changes in patient perceptions over time.

## Data Availability

The raw data supporting the conclusions of this article will be made available by the authors, without undue reservation.
